# Predicting Hospital Readmission for Campylobacteriosis from Electronic Health Records: A Machine Learning and Text Mining Perspective

**DOI:** 10.3390/jpm12010086

**Published:** 2022-01-10

**Authors:** Shang-Ming Zhou, Ronan A. Lyons, Muhammad A. Rahman, Alexander Holborow, Sinead Brophy

**Affiliations:** 1 Centre for Health Technology, Faculty of Health, University of Plymouth, Plymouth PL4 8AA, UK; 2 Health Data Research UK, Swansea University Medical School, Swansea University, Swansea SA2 8PP, UK; R.A.Lyons@Swansea.ac.uk (R.A.L.); S.Brophy@Swansea.ac.uk (S.B.); 3 Department of Computer Science, Cardiff Metropolitan University, Cardiff CF5 2YB, UK; mrahman@cardiffmet.ac.uk; 4 South West Wales Cancer Centre, Singleton Hospital, Swansea SA2 8QA, UK; alexander.holborow@Swansea.ac.uk

**Keywords:** hospitalisation, readmission, *Campylobacter* infections, machine learning, text mining, feature selection, electronic health records

## Abstract

(1) Background: This study investigates influential risk factors for predicting 30-day readmission to hospital for Campylobacter infections (CI). (2) Methods: We linked general practitioner and hospital admission records of 13,006 patients with CI in Wales (1990–2015). An approach called TF-zR (term frequency-zRelevance) technique was presented to evaluates how relevant a clinical term is to a patient in a cohort characterized by coded health records. The zR is a supervised term-weighting metric to assign weight to a term based on relative frequencies of the term across different classes. Cost-sensitive classifier with swarm optimization and weighted subset learning was integrated to identify influential clinical signals as predictors and optimal model for readmission prediction. (3) Results: From a pool of up to 17,506 variables, 33 most predictive factors were identified, including age, gender, Townsend deprivation quintiles, comorbidities, medications, and procedures. The predictive model predicted readmission with 73% sensitivity and 54% specificity. Variables associated with readmission included male gender, recurrent tonsillitis, non-healing open wounds, operation for in-gown toenails. Cystitis, paracetamol/codeine use, age (21–25), and heliclear triple pack use, were associated with a lower risk of readmission. (4) Conclusions: This study gives a profile of clustered variables that are predictive of readmission associated with campylobacteriosis.

## 1. Introduction

Campylobacteriosis is the most common form of culture-positive bacterial gastroenteritis worldwide, with the species C.*jejuni* and C.*coli*, inhabiting the intestinal tracts of both humans and animals, and accounting for up to 95% of human infections [1]. The disease burden has been estimated to be over 2.4 million people per annum in the USA [2,3]. In the UK, *Campylobacter* is thought to cause more than 280,000 cases of food poisoning annually, and be responsible for more than 100 deaths a year at an estimated cost of £900 million [4]. 

*Campylobacter* infections are typically attributed to the handling and consumption of chicken and, less frequently, with the consumption of unpasteurized milk, red meat, sausages, contaminated water, or transmission from household pets or farm animals. Most infections are sporadic, with relatively few identifiable outbreaks, so it is difficult to trace the sources and routes of transmission. Thus, translation of exposure to infection remains poorly understood [2,5,6,7,8]. 

Clinical manifestations of *Campylobacter* entirits typically include sudden onset abdominal pain, cramping, fever and frequent diarrhoea, with bloody stools in around one in ten patients. Fatality is most common in the elderly and those with comorbid conditions [9]. Late sequelae, such as inflammatory bowel diseases [10,11,12], rheumatologic disorders (i.e., reactive arthritis) [13,14,15,16], Guillain-Barré syndrome (GBS) [17,18], and Glomerulonephritis [19], often cause long term morbidity. In Europe, the incidence of campylobacter infection has continued to increase in the last decade, and reported increases in infection rates have necessitated the establishment of measures for prevention and control through the food chain [20]. Despite its high incidence, the factors associated with chronic infection or recurrence, and hence readmission, remain poorly understood.

Complications associated with *Campylobacter* infection often require hospitalisation [21,22,23], and in England and Wales approximately 10% of reported cases were admitted to hospital for treatment [24]. In an Australian provincial setting, the average anunual rate of Campylobacter-associated hospital admissions was 13.6%, and the readmission rate of Campylobacter-associated hospitalizaiton was 5.53% whthin 28 days after discharge [25]. In the USA, campylobacteriosis costs an estimated $1.3 billion a year in hospitalisation and other medical costs, surpassing salmonellosis and shigellosis [2,3], with unplanned readmission adding to the clinical and financial burden [26].

Readmission rates are utilised as indicators of hospital performance and quality of care. Absolute number and rate of readmission continue to rise in the UK, increasing by 19% between 2010 and 2017. Furthermore, readmission classified as potentially preventable is rising twice this rate, estimated at over 40% over the same time-period [27]. Preventable readmissions therefore represent an increasing burden on healthcare systems and hospitals have strong incentives to predict, at the time of discharge, patients who would be at high risk of readmission. The absence of effective predictive models currently limits the effectiveness of readmission reduction strategies. To develop a reliable predictive model, one first needs to identify modifiable predictors of readmission regarding patients and care. However, this can be challenging for diseases, such as campylobacteriosis studied here, where cases of infection are not well explained by the commonly recognized risk factors [6,28,29,30] and reliable predictors of hospitalisation have not been clearly established. 

In current clinical practice, the risk of patient readmission can be evaluated using the LACE index, defined by four independent variables: length of stay (L); acuity level of admission (A); comorbidity condition (C); and use of emergency rooms (E) [29]. Use of the LACE index, assuming a linear relationship among the four variables [30,31], can result in poor predictive performance [29]. In fact, there is no standard LACE threshold to classify patients as readmission versus non-readmission, and practitioner assessment is often subjective in defining such threshold. In contrast to the LACE index, some regression models have been developed to predict readmission from patient hospital records, but majority of the models [29,31,32] were not only built from a small number of variables but also were not developed to be generalizable, often relying on a small number of coded terms from primary care records. 

Following a decade of rising readmission rates in the UK, in 2011 the Department of Health introduced policies focussed on reversing this trend which included financial penalties for 30-day readmission [33]. This coincided with the US Hospital Readmission Reduction Programme, which also included punitive financial measures for underperforming hospitals [34]. The estimated cost of readmission in the USA stands at approximately $17.4 billion [33], unplanned readmission has therefore become a major concern in advanced healthcare systems. Procedures for reducing readmissions, such as education, follow-up visits, and discharge ‘teams’ have been implemented in many hospitals [34], but these methods are often impractical, costly and of limited impact. Indeed, readmission rates have continued to rise in English hospitals since the introduction of these policies [35]. In light of this, there is an urgent need to identify factors that accurately predict the risk of readmission.

Risk factors for campylobacteriosis are widely recognized [5,7,36,37] but reliable predictors of hospitalisation have not been clearly established. Recently natural language processing techniques were adopted to predicit hospitalizations with structural and unstructural data [38,39]. Here, we develop a robust, validated, and cost-effective data-driven method to identify the most informative hospital readmission predictors from primary care medical records. We use a novel incorporation of machine learning techniques with electronic health records, in which clinical terms (diagnosis codes, procedure codes and medication codes) recorded in general practice were analysed using a text mining scheme, while the prediction of the re-hospitalisation was treated as a problem of document classification in text mining.

## 2. Materials and Methods

**Figure 1:**
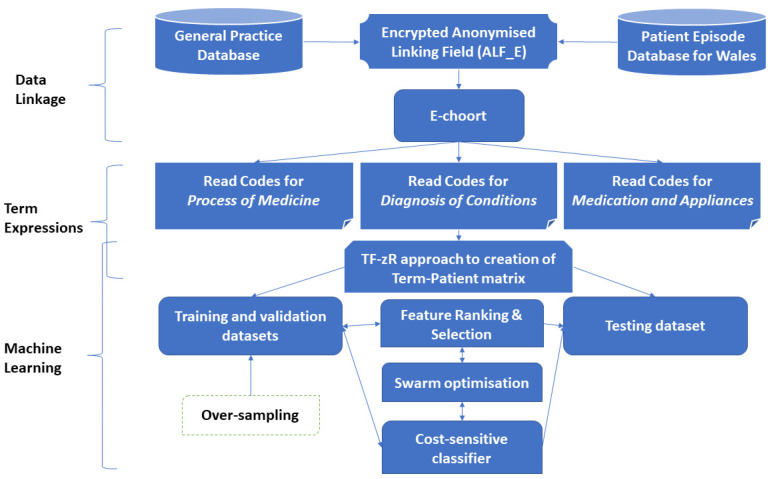
The flow diagram of the process of building readmission prediction model.

This study aims to identify key influential factors from a pool of demographic variables and clinical events recorded in primary care to predict the outcomes of campylobacteriosis patients admitted to hospital, classified as ‘*readmission*’ and ‘*non-readmission*’. Figure 1 illustrates the process of building the prediction model.

### 2.1. Data Collection and Linkage

A GP database from the Abertawe Bro Morgannwg University (ABMU) Health Board area with *Campylobacter* infections between 1990 and 2015 was linked to the Patient Episode Database for Wales (PEDW) which records all episodes of inpatient and daily case activity in NHS Wales hospitals. The data linkage was conducted via the Secure Anonymised Information Linkage (SAIL) databank [40,41]. The SAIL databank brings together and links a wide range of person-based data from multiple sources relevant to health. SAIL utilises a range of measures to ensure that the data are anonymous and secure, and that they can be safely utilised for research within a robust information governance framework [41]. Each patient was given a unique Encrypted Anonymised Linking Field (ALF_E). Different databses of electronic health records hold inidviudal ALF_Es which indicate patient level data records. So, these different databases can be linked at individal level by the ALF_Es.

In this study, 12,747,826 rows of electronic health records for all patients with *Campylobacter* infections held in the GP database were linked with the hospital admission database by the ALF_E. The patients with *Campylobacter* infections, defined in terms of Read code (“*A0473*” for *Campylobacter* gastrointestinal tract infection) and in GP data and ICD 10 code (*A045—Campylobacter* enteritis in hospital admissions), were extracted and the date of first occurrence was selected. All admissions took place after an infection. The inclusion criteria for GP records were (i) the patient was alive at discharge and (ii) the patient was enrolled within the GP record for 12 months before the date of infection. For each patient, within 12 months after Campylobacter infection, readmission was defined as any admission taking place within 30 days following discharge from the previous admission. This readmission was regarded as a reference admission. If there was no 30-day readmission, the first admission was treated as a reference admission. In other words, for each patient, their GP records from 12 months before the infection to their reference admission were collected. If a patient was readmitted on the same day of discharge, it was counted as a single continuous admission. For patients who had multiple *Campylobacter* infections leading to different hospital admissions, some infections may lead to readmission, others did not. In this situation, each infection was treated separately, as they corresponded to different GP visit records. In this way, a total of 13,006 patients admitted to hospital with Campylobacter infection were obtained, 8.17% of which (1062) were readmissions. In other words, these 13,006 patients generated 12,747,826 rows of records in the GP databse.

Additional demographic variables included: deprivation status in terms of Townsend score (quintile); urban status as defined by the Office for National Statistics [42]—‘*Urban* > *10 k* (*Urban Settlements with greater than 10 k population)*’, ‘*Town and Fringe (located within the rural domain)*’, ‘*Village*, *Hamlet and Isolated Dwellings(located within the rural domain)’*; age bands between GP event date and date of birth (0–5, 6–10, 11–15, 16–20, 21–25, 26–30, 31–35, 36–40, 41–45, 46–50, 51–55, 56–60, 60+). Thus, the initial variables were formed by gender, age groups and bands of deprivation, and medical records held electronically in general practice and in the hospital admission dataset.

The 13,006 hospital admitted patients were further randomly split into training (70% of the data), testing (15%) and validation (15%) data subsets for constructing machine learning models, selecting the optimal model and testing performance respectively.

### 2.2. Machine Learning Approach

This cohort study used machine learning methods to identify influential risk factors that are most predictive of readmission of *Campylobacter* infections from routine electronic health records. However, the linked dataset includes different health-related fields with a range of data structures, for example, age and deprivation fields are categorical values, while the majority of the factors are text terms from the GP database based on NHS Read Clinical Term system. The general practice system with 5-bytes provides around 83,000 clinical descriptive terms in hierarchical structure comprising five levels of detail, whilst each successive level offers more detail to a concept. This means, there are multiple codes for the same medication/diagnosis/procedure with a progressive level of detail. Such heterogeneous linked data presents methodological challenges for predictive analytics [43,44]. To address the challenges, we integrated machine learning and natural language processing (NLP) techniques to identify the most influential predictors associated with the readmission of *Campylobacter* infections from the large number of heterogeneous variables. A ‘bag of words’ (BoW) scheme [45] consisting of coded terms and other variables (words) was used to describe each patient, where the number of occurrences of each term was recorded. The prediction of readmission for each patient was thus treated as a problem of text classification. The proposed methodology is described below.

First, a *Term-Patient* matrix was created to represent each patient by the BoW of terms. Traditionally, the term frequency (how often a term occurs) was used to weight each term, for example, a blood pressure check may happen five times a year, a diagnosis may happen once. However, text mining studies have indicated that term frequency (TF) based classification methods often fails to effectively distinguish the individuals (patients here) [45]. Thus, in this paper, an approach called *TF-zR* (term frequency-zRelevance) technique was devecloped to evaluates how relevant a clinical term is to a patient in a cohort characterized by coded electronic health records (see Appendix A). The *zR* is a supervised term-weighting (STW) metric to assign a weight to a term based on relative frequencies of the term across different classes (i.e, readmission and non-readmission). The mechanism behind such a STW method is that the more relevant term should be the one with more concentrated frequency in one class (positive class: readmission/negative class: non-readmission) than the other class. Then the TF value and the STW metric were combined to represent each patient (see Appendix A). In this way, a quantitative digest of each patient represented by this *TF-zR* method in a *Term-Patient* matrix was obtained. This method is different from traditional unsupervised term-weighing methods which do not consider the impact of sample distributions across different classes.

For this *Term-Patient* matrix, each variable was then ranked using the information gain method [46] (see Appendix A) to examine its capacity of distinguishing readmission with non-readmission across all patients. Normally, the Read codes in general practice fall into the categories of “*Process of Medicine*” (PoM) (such as laboratory tests), “*Diagnosis of Conditions*” (DoC) and “*Medication and Appliances*” (MaA). The issue is that the PoM Read terms are frequent but carry less information, while the DoC and MaA terms, such as a diagnosis of diabetes, may occur once in a patient’s lifetime but are important and carry more information. Therefore, it is unsurprising that using a purely TF method, the PoM codes will suppress the impact of DoC and MaA, although the latter provide more meaningful clinical knowledge of patient’s health conditions in terms of diagnoses and treatments. To avoid the suppression of the PoM codes, in this study the Read codes in each category were assessed and ranked separately in terms of their capacity of distinguishing the outcomes. Then along with demographic variables, the pool of the selected codes from each category were used for constructing a classification model in the next phase.

Of the 13,006 patients admitted to hospital with *Campylobacter* infections, there were only 8.2% readmissions. Thus, this is an extremely imbalanced data problem. To address the problem, a cost-sensitive classification scheme [47,48] is used to provide different penalties of misclassifications of readmission and admission. Specifically, the cost of misclassifying readmission as admission is greater or more serious than misclassifying admission as readmission. Using particle swarm optimisation [49] and a weighted learning scheme, the model with the most influential predictive factors was then identified, offering the best potential of distinguishing those that were readmitted to hospital with those that were not. The read codes in the categories of DoC and MaA were given higher weighting than those in PoM. The final selected predictors were then validated with the independent unlabelled samples in a testing data subset.

The performance of the model was assessed in terms of sensitivity and specificity against 15% of the total database.

To further validate the performance of the identified clinical and demographic signals in predicting the hospitalization of *Campylobacter* infection, the over-sampling technique was used to adjust the class distribution of the trained data set for building machine learning models with the more balanced data set.

## 3. Results

This study utilised 12,747,826 health records of 13,006 patients admitted to hospital with *Campylobacter* infections between 1990 and 2015, while there were 1062 readmissions. So, this is a highly imbalanced data problem where the negative class has much more samples than the positive class. Table 1 shows a demographics table of *Campylobacter* infection admissions. Children aged 0–5 had the highest rates of hospital admissions, while patients aged between 46 and 55 had the highest rates of readmission. Children aged 6–15 had the fewest overall hospital admissions and readmissions. Due to a denser population, more people living in the urban areas had hospital admissions and readmissions than those living in town and fringe, or village, hamlet and isolated dwellings. Among patients in the 5th Townsend deprivation quintile (the most deprived), the rate of their re-admissions was 8.66%, higher than the rate of re-admissions (7.92%) among those in the 1st deprivation quintile (the most affluent). It is noted that although these statistics showed the overall impacts of demographic factors on hospital admissions, this does not mean that they are significant in predicting the readmissions as the predictors also depend on interactions between variables.

There were 23 categorical demographic variables generated on gender, age groups, deprivation and urbanicity. In addition, 17,483 clinical events were classified by read codes into the categories of PoM (8206 codes), DoC (3702 codes) and MaA (5575 codes). In this way, the linked dataset generated initial data with 17,506 variables. These variables were taken forward to identify the most influential predictors associated with the Campylobacter readmission.

**Figure 2:**
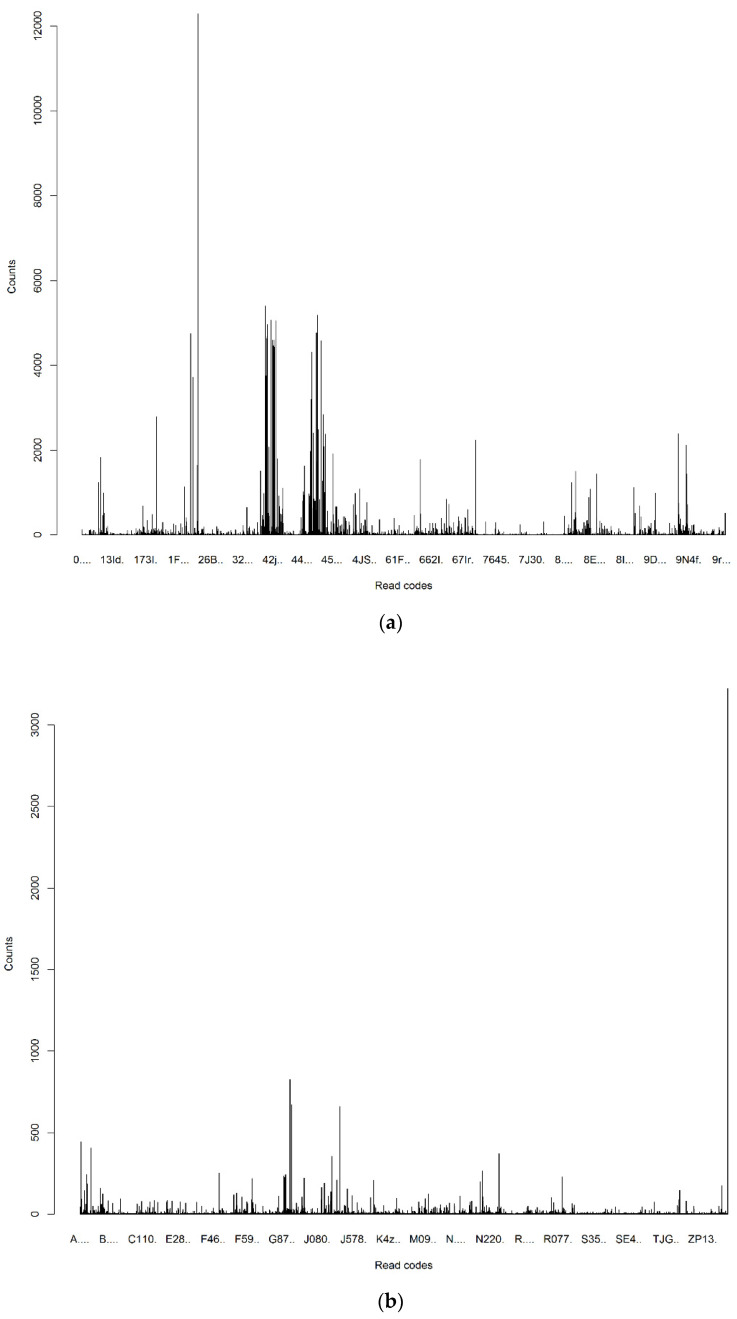

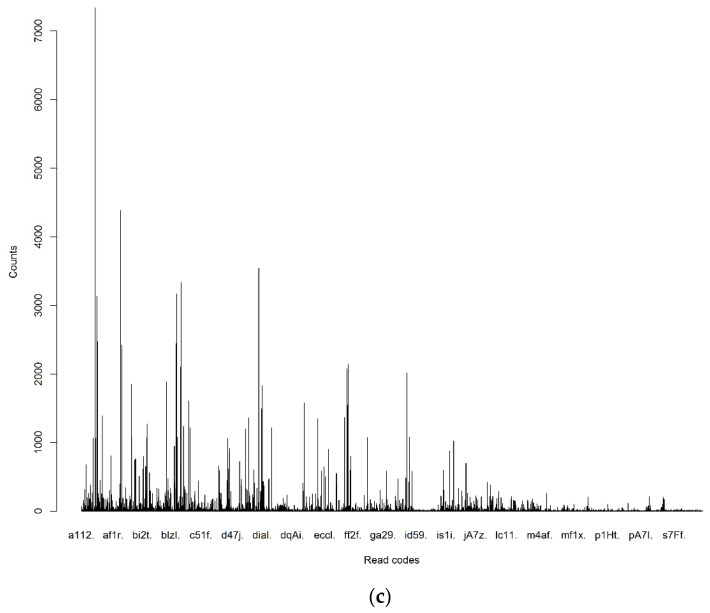
Frequencies of read codes in different categories. (**a**) Frequencies of read codes in PoM; (**b**) frequencies of read codes in DoC; and (**c**) frequencies of read codes in MaA.

**Figure 3:**
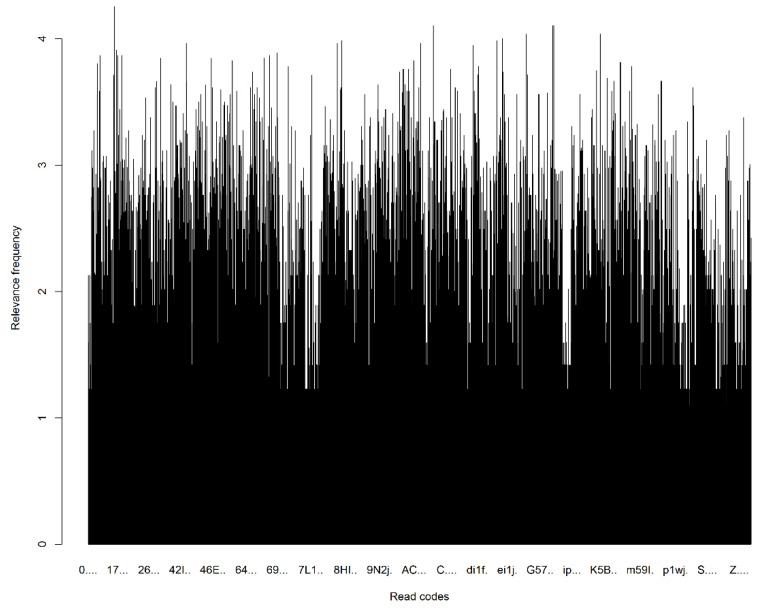
The supervised term-weighting metric.

These clinical terms demonstrated a great disparity in term of TF across the different categories of PoM, DoC and MaA (Figure 2). The wide range of frequency variations exactly characterises real clinical practices, in which the PoM events often occur much more frequently than those of the DoC and MaA. It is noted that these frequency measurements did not take into account their relevance to the re-admission. Differently, the supervised term-weighting method—zR metric took into account the contributions of a term across different classes (re-admission and non-readmission) to generate the relevance measurements which show much better proportionality for different categories of read codes (Figure 3).

**Figure 4:**
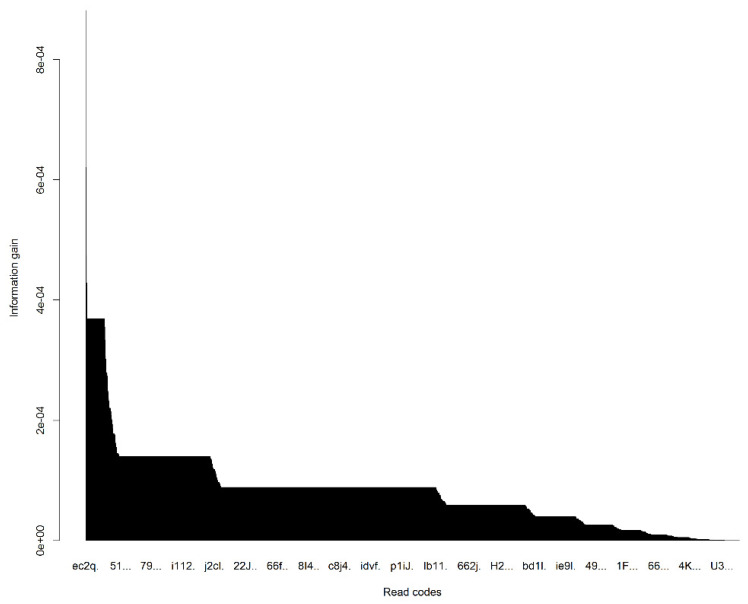
The ordered information gains of variables in distinguishing between the readmissions and admissions.

After the *TF-zR* method generated the *Term-Patient* matrix, applying information-gain to this *Term-Patient* matrix allowed the generation of a feature ranking metric for each variable which assessed the contribution of each variable in distinguishing between the readmission and non-readmission (Figure 4). Information-gain is normally used to determine the influential features/attributes/variables that render maximum information about a class. So in terms of information-gain, the top read codes were selected from each category of PoM, DoC, and MaA. Then together with 23 categorical demographic variables, a data space with total 623 variables was generated. From these 623 variables, the swarm optimization with weighted subset learning and cost-sensitive decision tree classifier identified the 33 optimal features that offered the best potential of predicting the hospitalisation of *Campylobacter* infections (Table 2). The 33 most predictive variables included an age group (ages 21~25 associated with non-readmission), gender, Townsend deprivation quintiles (bands 1 and 4), comorbidities (12 diagnostic codes), medications (11 prescription codes) and procedures (6 codes). Applying to an independent test dataset, the classifier with the 33 influential predictors performed significantly above chance to predict readmissions with sensitivity 0.73 (95% confidence interval (0.71, 0.75)), and specificity of 0.54 (95% confidence interval (0.53, 0.55)). Cystitis, paracetamol and codeine use, age (21 to 25), and heliclear triple pack, have turned up to be very efficient in classifying the outcomes of *Campylobacter* infections, where patients with these conditions had lower risk of readmission.

To further validate the performance of the 33 predictors in this imbalanced data problem, we applied to an independent balanced dataset produced by the over-sampling technique, the 33 predictors predicted readmissions with sensitivity 0.91 (95% confidence interval (0.90, 0.913)), and specificity of 0.54 (95% confidence interval (0.52, 0.565)).

In order to demonstrate the efficiency of how the developed modelling approach tackles the issue of imbalanced classes in medical data, we further compared with logistic regression, a traditional modelling approach to readmission prediciton. Using the raw data with same training and testing datasubsets as our model, the logistic regression model with the same 33 influential predictors offered prediction of readmissions on testing datasubset with sensitivity 0.0298, and specificity 0.974. Clearly logistic regression approach cannot tackle the imblanced data issue. Then using the same oversampled data as our model, the logistic regression model with the same predictors significantly improved the prediciton performance with sensitivity 0.8196, and specificity 0.5253, but still compared unfavourably with our developed modelling approach.

## 4. Discussion

By integrating text mining, feature selection, and machine learning, our study provides a novel methodology for building a predictive model capable of automatically identifying influential risk factors from primary care records with good predictive performance.

Using this methodology, we identified 33 most predictive variables of age, gender, deprivation, comorbidities, medication and medical procedure. Analysis of the clinical implication of these variables revealed that most of the predictors of readmission relate to comorbidities of recurrent minor illness (e.g., recurrent tonsillitis, non- healing open wounds, ingrown toenails, impacted cerumen (wax in ear)). Males with a history of recurrent minor illnesses are at increased risk of readmission, indicating that patient profiling could help with support at discharge and more targeted use of antibiotics. Each such condition may not be directly important in the outcomes of *Campylobacter* infection, but combined, they give a profile of individuals that have a history of chronic minor illness and may be less well equipped to take care of themselves. These ‘at risk’ patients may require additional support at discharge to reduce readmission risk. Such support could include enhanced patient education during discharge, conducting follow-up visits or medication reconciliation [50]. These ‘at risk’ patients contrast with the profile of patients least likely to be re- admitted, typically younger females with a history of seeking treatment for bacterial infections and taking medication for illness. Cystitis has emerged in our study as the most effective variable in predicting no readmission for the campylobacter. Campylobacter infection patients with cystitis had a lower risk of readmission once they were discharged. Perhaps this signals the profile of the person with the least chance of readmission is more likely female and reports bacterial infections. The predictions identified in this study therefore provide a justification for using comorbidity as an indicator in the LACE index as assessed by *Charlson* comorbidity index to predict readmissions.

There are several advantages to the machine learning approach employed in this study. First, it works efficiently with a large and very high dimensional dataset for developing predictive models, which allows the predictive models to avoid the challenges of dimensionality [51]. Second, most machine learning algorithms fail to work with imbalanced datasets due to subject to a frequency bias in which more emphasis is placed on learning data observations with more occurrences. Our methodology integrates a cost-sensitive learning scheme to effectively identify the influential factors. Third, different from classic unsupervised term-weighting methods including frequency, our methodology used a supervising term weighting method to generate patient representations by considering the disparity of term distributions across data classes. This provides a foundation for identifying predictive factors with good capacity for distinguishing the outcomes of health conditions. Fourth, different from existing readmission predictive models without considering model generalisation performance during construction, our methodology centred on generalisation performance of the constructed model by adopting optimal model selection scheme and using independent data subsets for the different purposes of model constructions, hyper-parameter identification and model evaluation.

However, the proposed methodology has some limitations. It requires a high computing load to build a robust prediction model, and extensive cross-validation to evaluate the potential predictors identified. Furthermore, there are variations unexplained by this prediction model and additional information about the infections (strain, severity) and the symptoms are needed to improve the prediction performance.

This study was developed with a focus on campylobacter infection related admissions, future studies should explore the usability/fittingness of such machine learning and state-of-the-art methods of natural language processing, such as transformer models such as BERT [52], BioBERT [53], for word representations in readmission prediction.

## 5. Conclusions

By identifying predictors of readmission for campylobacter infections in primary care setting, we conclude that patients with a history of recurrent minor preventable illnesses may need greater support upon discharge from hospital to prevent readmission. This is important for reducing the burden on secondary care services that readmission represents and in improving care for patients. The effectiveness of this approach demonstrates the potential in machine learning methods in adopting personalised medicine to meet the goal of reducing preventable readmissions.

## Figures and Tables

**Table 1:** Demographics table of campylobacter admissions.

	Non-Readmission	Readmission
Number of admitted patients	11,944	1062
Average age of admissions	43.0 (SD = 22.1)	51.8 (SD = 22.3)
Percentage of male admissions	49.9%	55.6%
Percentage of admissions in most affluent	22.4%	21.7%
Percentage of admission in most deprived	17.6%	18.7%
Percentage of admissions in rural domains *	37.2%	37.7%

* Including “Town and Fringe (located within the rural domain)”, and “Village, Hamlet and Isolated Dwellings (located within the rural domain)”.

**Table 2:** The 33 key predictive variables identified.

Variable Name	Description
Male	Gender
AGE_A21–25	Age group: 21~25 years old
TOWSEND_Q1	Townsend quintile band 1
TOWSEND_Q4	Townsend quintile band 4
67E..	Foreign travel advice
8B311	Medication given
67H..	Lifestyle counselling
9....	Administration
8H7..	Other referral
7G300	Excision of nail bed
R0901	Abdominal colic
K3108	Breast infection
S89z.	Other open wounds NOS
K15..	Cystitis
H170.	Allergic rhinitis due to pollens
H037.	Recurrent acute tonsillitis
A53..	Herpes zoster
N05..	Osteoarthritis and allied disorders
Ayu03	Salmonella infection, unspecified
F501.	Infective otitis externa
F504.	Impacted cerumen (wax in ear)
N2410	Myalgia unspecified
dian.	Paracetamol+codeine phosphate 500 mg/30 mg tablets
ka91.	Celluvisc 1% single-use eye drops
da7Z.	Venaxx XL 75 mg m/r capsules
dher.	Prochlorperazine 5 mg tablets
c13M.	Ventolin 200 micrograms Accuhaler
bs18.	Warfarin sodium 3 mg tablets
a6g2.	Heliclear triple pack
k3g1.	Fusidic acid 1% eye drops
e91E.	Erythromycin 125 mg/5mL sugar free suspension
c61z.	Beclometasone dipropionate 100 micrograms inhaler
da61.	Paroxetine 20 mg tablets

## Data Availability

Data are available from the SAIL (Secure Anonymised Information Linkage) Databank for researchers who meet the criteria for access to confidential data.

## Appendix A

### Appendix A.1. zR: Supervised Term Weighting Metric

As each patient is described by a series of read codes and additional categorical demographic variables, these codes and variables can be treated as terms as carried out in the text mining. In this way, mining electronic health records in primary care and secondary care settings corresponds to the task of text categorization which automatically classify textual documents into different predefined semantic classes. Certainly, different terms in a document (i.e., a patient record here) often make different contributions to the semantics of the document. Term weighting is an important step to assess the importance of terms in classifying unlabelled natural language documents.

In this study, we consider a term weighting scheme by which the more important term should be the one with more concentrated occurrence in one class (positive class/negative class) than the other class (negative class/positive class). To formalize this idea, we use *a*, *b*, *c*, and *d* to denote the number of different patient records, as listed below:

- *a* = the number of patient records in the Class 1 that contains the term *t*.
- *b* = the number of patient records in the Class 1 that does not contain the term *t*.
- *c* = the number of patient records in the Class 0 that contains the term *t*.
- *d* = the number of patient records in the in Class 0 that does not contain the term *t*.

where the Class 1 is the category of patients re-admitted to hospital, the Class 0 is the category of patients not re-admitted to hospital. Then we can have a contingence table of term *t* across the two classes of patients (see Table A1).

**Table A1:** Contingence table of term *t* across the two classes of patients.

	Term t		
	Yes	No	Sum
Class 1	a	b	$N_{1.}$
Class 0	c	d	$N_{0.}$
Sum	$N_{.1}$	$N_{.0}$	*N*

where

N_.1_ = *a* + *c*, N_.0_ = *b* + *d*, N_1._ = *a* + *b*, N_0._ = *c* + *d*, N = *a* + *b* + *c* + *d*.

Then we first have the information gain of the term *t* defined as

$$ig=\frac{a}{N}\cdot log\left(\frac{a\cdot N}{N_{.1}\cdot N_{1.}}\right)+\frac{b}{N}\cdot log\left(\frac{b\cdot N}{N_{.0}\cdot N_{1.}}\right)+\frac{c}{\;N}\cdot log\left(\frac{c\cdot N}{N_{.1}\cdot N_{0.}}\right)+\frac{d}{N}\cdot log\left(\frac{d\cdot N}{N_{.0}\cdot N_{0.}}\right)$$

where the base of this logarithmic operation (*log*) is 2. Information gain computes the he impurity in class elements by following the concept of entropy while aiming at decreasing the level of entropy.

We propose *a* supervised term weighting-based relevance metric, zRelevance (*zR*), to describe the situation of the term *t* which occurs more often in one class than in the other class.

$$zR=p_{1}^{t}\cdot \log\left(2+\frac{a}{\max\left(c,1\right)}\right)+p_{0}^{t}\cdot \log\left(2+\frac{c}{\max\left(a,1\right)}\right)$$

where $p_{1}^{t}$ and $p_{0}^{t}$ are the relative frequencies (probabilities) of term t occurring across the patient records of different classes:

$$p_{1}^{t}=\frac{a}{N_{.1}}$$

$$p_{0}^{t}=\frac{c}{N_{.1\;}}$$

The weight for the term *t* is finally assigned as

W(t)=zR

### Appendix A.2. TF-zR Approach to Creation of Term-Patient Matrix

Assuming there be m patients and n terms in the database (i.e., each patient is characterized by these n terms). Let $tf_{ij}$ represent the frequency of the term $t_{j}$ in the records of the patient $pt_{i}$. Then the *Term-Patient* (*TP*) *matrix* used to represent the relationships between clinical terms and outcomes can be generated as

$$TP_{ij}=tf_{ij}\cdot W\left(t_{j}\right)$$

where $W\left(t_{j}\right)$ is the zR relevence metric defined above.
